# The use of flipped classroom as an active learning approach improves academic performance in social work: A randomized trial in a university

**DOI:** 10.1371/journal.pone.0214623

**Published:** 2019-04-04

**Authors:** Bárbara Oliván Blázquez, Barbara Masluk, Santiago Gascon, Ricardo Fueyo Díaz, Alejandra Aguilar-Latorre, Isabel Artola Magallón, Rosa Magallón Botaya

**Affiliations:** 1 Department of Psychology and Sociology, Faculty of labour and social sciences, University of Zaragoza, Zaragoza, Spain; 2 Institute of Health Research of Aragon (IIS), Zaragoza, Spain; 3 The Research Group B21_R17 of the Department of Research, Innovation and University of the Government of Aragón, Zaragoza, Spain; 4 Education council of the board of the Autonomous Community of Madrid, Madrid, Spain; University of Westminster, UNITED KINGDOM

## Abstract

**Background:**

The flipped classroom (FC) is a pedagogical approach that means that the activities that have traditionally taken place within the classroom are carried out outside the classroom. Fundamentally it implies the way in which the student studies the subject. This change of perspective in teaching—learning has raised many questions regarding its effectiveness and student satisfaction in the university studies in the degree of Social Work.

**Objective:**

The main objective of this study was to evaluate the effectiveness of a Flipped Classroom methodology in the academic performance of students of the Social Work Degree.

**Methods:**

An educational study, with two parallel groups was developed. The randomization was carried out by class groups. Group 1 was assigned an active teaching methodology of Flip Teaching and it was implemented during theoretical teaching hours. The other group of students, Group 2, was assigned a traditional lecturer-based learning (LB) methodology. The participants were all the students of the morning shift who studied the subject Social Work with Groups of the Social Work Degree during the academic year 2017–2018. The sample was composed of 110 subjects, with 60 subjects who developed an active teaching methodology and 50 subjects who received a LB.

**Results:**

In terms of the academic performance result variable, the FT group had a mean of 6.56 (SD: 1.58) and the LB group had a mean of 5.42 (SD: 1.97) (p-value: 0.002). The FT group also had a higher percentage of students receiving merit and outstanding scores (34.5% and 6.9% respectively) and a lower percentage of students who failed (19%) as compared to the LB group in which 20.9% and 2.3% of the students received merit or outstanding grades and 46.5% failed (p-value = 0.025). No significant differences were found with regards to satisfaction with the subject and the methodology used, long-term learning and time spent preparing for the exam.

**Conclusions:**

The FC teaching methodology in comparison with the LB methodology has shown to be a more effective tool regarding academic performance evaluated in a quantitative and qualitative way with regards to Social Work education at university level.

## Introduction

In Europe, higher education has been modified in the last decades thanks to the creation of the European Higher Education Area (EES) developed through the Bologna Declaration [1], the Prague Declaration [2], the European Union Council Meeting [3] and the Lisbon Declaration [4]. The European Higher Education Area transfers the emphasis in the teaching-learning process from a model of knowledge transfer by teachers to a learning model based on student-centered competences. Therefore, it has been necessary to include active learning methodologies that entail a greater degree of involvement on the part of the student, a greater dynamism in learning and a greater interaction with the contents [5–6].

An active learning methodology is the so-called Flipped Classroom (FC), which is consistent with a competency-based model [7].

According to the principles of FC teaching methodology, learning is at the center of the educational process, the student having a very active role, with the teacher guiding and facilitating the learning process. Fundamentally it implies the reorganization of activities according to the place where they are executed, that is, what was traditionally taught in the classroom, as this methodology is used outside the classroom.

Recent studies [8,9] consider FC as a pedagogical model that includes interactive and group learning activities within the classroom, since it is in this place that the processes of acquisition and practice of theoretical knowledge are enhanced. Another component is the transfer of individual learning outside the classroom. In fact, authors such as Chen et al. [10] define FC as a hybrid approach that combines on-line autonomous learning and face-to-face activities in the classroom. The students engage in content learning before class, thereby maximizing in-class time for active learning.

Although modern versions of FC appeared over ten years ago, it is generally popular in education, higher education and specially sciences and medical education [11]. Over the last few years studies of the effectiveness of FC in the training of medical degrees in various areas including [12–15], pharmacy [16–17], nursing [18–21], physiotherapy [22], veterinary [23], chemistry [24], engineering, and physics [25–27], have been developed.

The studies carried out on the effectiveness of FC in the field of social sciences [28–29]and specifically in social work degrees are more scarce [30–31].

Innovation in the education of social work, which encompasses multiple functions including teaching, research, and practice, is constrained by the limits of current social work approaches and methods. Increasing social work's impact in the real world requires the clarification of the complexities of reciprocal forces between human lives and the environment [32].

This means that innovative and active teaching methodologies can be developed through reflective journalism [30], readings accompanied by online tools that promote learning communities and prompt further reflection, etc. [31].

The FC method has shown greater academic achievement than traditional lecturer-based learning (LB), and this fact has been more evident in recent years [10] mainly due to the development of technological resources such as Google Drive, YouTube, Vimeo, Google Classroom, etc. [7]. However, results should be interpreted with caution because of the high methodological diversity, statistical heterogeneity and risk of bias in the studies carried out. It has been stated that future studies should have higher methodological rigor, a standardized FC format and utilize assessment tools evaluating higher cognitive learning process instead of an exam [10].

Given the scarcity of work in social sciences and specifically in SW, and the heterogeneity of studies published with high risk of bias, the development of a study that provides scientific evidence through a randomized trial methodology, thereby minimizing the risk of bias, is proposed.

The main objective of this study is to evaluate the effectiveness of a Flipped Classroom methodology in the academic performance of students of the Social Work Degree.

The aim is also to evaluate the effectiveness of FC regarding satisfaction with the subject and the methodology used, with respect to the hours of study spent on preparation for the exam, with respect to the short-term learning of each theoretical topic and, lastly, with respect to the perceived difficulty of each theoretical topic.

The alternative hypotheses for this study are the following:

H1: Students who develop an active methodology of FC-based learning obtain higher levels of academic performance, valued both quantitatively and qualitatively with respect to students who develop a traditional methodology.
H2: Students who develop an active methodology of FC-based learning are more satisfied with the subject and the methodology used with respect to students who develop a traditional methodology.
H3: Students who develop an active FC-based learning methodology spend less time preparing for the exam than students who develop a traditional methodology.
H4: Students who develop an active FC-based learning methodology acquire more short-term learning from each theoretical topic compared to students who develop a traditional methodology.
H5: Students who develop an active FC-based learning methodology acquire greater long-term learning from each theoretical topic compared to students who develop a traditional methodology.
H6: Students who develop an active FC-based learning methodology regard each theoretical topic as less difficult when compared to students who develop a traditional methodology.

## Material and methods

### Design of the study

An educational study (randomized trial), with two parallel groups was developed. The randomization was carried out by class groups. Group 1 was assigned an active teaching methodology of Flip Teaching and it was implemented during theoretical teaching hours. The other group of students, Group 2, was assigned a traditional methodology, a master class. These methodologies were developed for the subject *Social Work with Groups*, in the Social Work degree at the University of Zaragoza (Spain).

The Social Work degree at the University of Zaragoza consists of 240 ECTS credits, distributed over four years. It includes core, compulsory, optional and external practice courses (presential and research), as well as an Undergraduate Dissertation. In the core, compulsory and optional courses consisting of 5 or 6 ECTS, there are some hours of theoretical training, practical education and a mentored project. The study plans may be viewed at the following link: https://estudios.unizar.es/estudio/asignaturas?anyo_academico=2018&estudio_id=20180110&centro_id=108&plan_id_nk=274&sort=curso.

The Social Work with Groups course is a compulsory course offered during the second quarter of the second course year. It includes two parts: a first part that is taught from a social psychology perspective, consisting of group psychology (five subjects of the course curriculum); and a second part that is taught from a Social Work and Social Services perspective, which specifically covers the handling of groups from the social work perspective (four subjects of the course curriculum). The bibliography used for the course may be viewed at the following website: http://psfunizar7.unizar.es/br13/egAsignaturas.php?codigo=26115.

### Participants and sample size

The population consisted of students registered in the Social Work with Groups course at the University of Zaragoza (Spain) during the 2018–2019 course year. During this academic year, 171 subjects were registered and 160 attended the theoretical and practical sessions on an ongoing basis. Given the population size of 160 subjects, assuming an error of 5% and a probability of success of 95%, with a confidence level of 95% and a precision of 5%, and adding 10% for potential abandoning of the intervention, at least 56 students were necessary. Since the intervention was conducted by class groups, 110 students participated in the study, exceeding the necessary sample size.

The participants were all the students of Group 1 and 2 of the morning shift who studied the subject *Social Work with Groups* of the Social Work Degree and who attended continuously both the theoretical and practical classes during the academic year 2017–2018. The sample was composed of 110 subjects, with 60 subjects who developed an active teaching methodology and 50 subjects who received a master class methodology. Randomization by cluster was carried out by means of a randomization computer program by an independent investigator in the study.

### Interventions

The teaching methodology was developed over six weeks during the months of February and March of 2018, during which five subjects of the course curriculum that belong to the area of knowledge of Social Psychology are taught. These topics address the following contents: 1) definition and types of groups; 2) group development processes; 3) group structure; 4) leadership; and 5) other aspects of groups—communication, empathy, obedience. There were four teaching hours a week, two of theoretical content and another two hours of practical content, and one hour of tutored group work.

The intervention was carried out in the hours dedicated to the theoretical content. The same teacher was used for both groups and performed the same activities in the practical classes and in the tutored group work. This teacher has five years of teaching experience in this subject.

In the intervention group where the Flip Teaching methodology was implemented, the students had to work on the contents previously to the theoretical classes that were to be taught.

To ensure this methodology was well-accepted among the students, the recommendations of Rotellar & Cain [33] were followed. To do this, the students were provided with written documentation and videos prepared through the Active Presenter program by the teacher of the subject.

Both the documentation and the videos were about each of the topics that were taught. In addition, the students had to answer questions that the teachers raised regarding each topic. During class time, the discussion was about the answers to the questions that had been answered outside of class time.

In the control group a traditional lecturer-based learning (LB) was developed, consisting of the teacher explaining the theoretical contents in the class without any previous work by the students on those theoretical contents.

In order to establish that both groups had the same teaching load throughout the six weeks that the teaching was given, ten teaching hours of theoretical class were established for the group that followed the FC teaching methodology, since they were working at home, and twelve teaching hours of theoretical class were established for the control group.

### Measurement instruments

#### Primary outcomes

The outcome variable of this educational study was the academic performance, evaluated by the grade obtained in a theoretical exam on the subject. This exam consisted of 35 multiple-choice questions with three response options, taking into account the chance factor (so the erroneous answers were discounted in the grade). The exam contained nine questions referring to group definition and types; seven questions referring to group development processes; nine questions referring to group structure; seven questions referring to leadership; and three questions referring to other group aspects—communication, empathy. The quantitative rating can range between 0 and 10, with a higher score denoting a higher percentage of success in the answers. The qualitative grading is *fail* (between 0 and 4.9), *pass* (between 5.0 and 6.9), *merit* (between 7.0 and 8.9) and *outstanding* (between 9.0 and 10).

#### Secondary outcomes

The secondary variables were: Student satisfaction with the subject and teaching methodology used, approximate number of study hours in the preparation for the exam, short-term learning of each subject taught and perceived difficulty of each topic.

To measure the secondary variable "student satisfaction with the subject and the methodology used", a self-made questionnaire consisting of seven questions was used, which are answered on a Likert scale from 0 to 4, with 0 being *not at all* and 4 being *to a great extent*. The questions were as follows: the methodology used has promoted new knowledge; it has favored a deep learning; it has helped me to think more critically; it has helped me to apply theoretical content to practice; it has helped me to apply theoretical content to evaluation; it has helped me to better understand concepts; and I think it is a good teaching methodology. An open section was also considered so that students could express their opinion freely. Another secondary variable that was collected was the number of approximate study hours spent in preparation for the exam. To measure "the short-term learning of each subject taught" secondary variable, the teacher proceeded as follows: at the end of each theoretical topic, a small test was carried out with between five and ten questions, depending on the size and difficulty of each topic, with four answer options, to assess the learning accomplished on the subject. This learning variable at the end of each topic was assessed on a scale between 0 and 10, with a greater number of correct answers giving a higher score. The computer application *Kahoot* was used to evaluate this variable.

The "long-term learning" variable was also measured. To do this, the students were summoned eight months later to take a test on the subject. This was asked through the class delegates, one day in advance, requesting their participation, explaining the purpose of the activity, and asking that they not review the subject as it was a test to assess their memory. A test of ten test questions was developed, with three response options. Only the correct answers were counted. These 10 questions were different from those responded to in the course assessment, corresponding to two questions per each of the 5 areas taught: 1. group definition and types; 2. group development processes; 3. group structure; 4. leadership; and 5. other group aspects—communication, empathy, obedience and group thinking. Questions referred to the basic and fundamental aspects of each of these areas.

Finally, at the end of each theoretical topic, the “perceived difficulty” variable in understanding the topic was also measured on a scale of 0 to 10, with 0 being *not at all difficult* and 10 *extremely difficult*.

**Fig 1:**
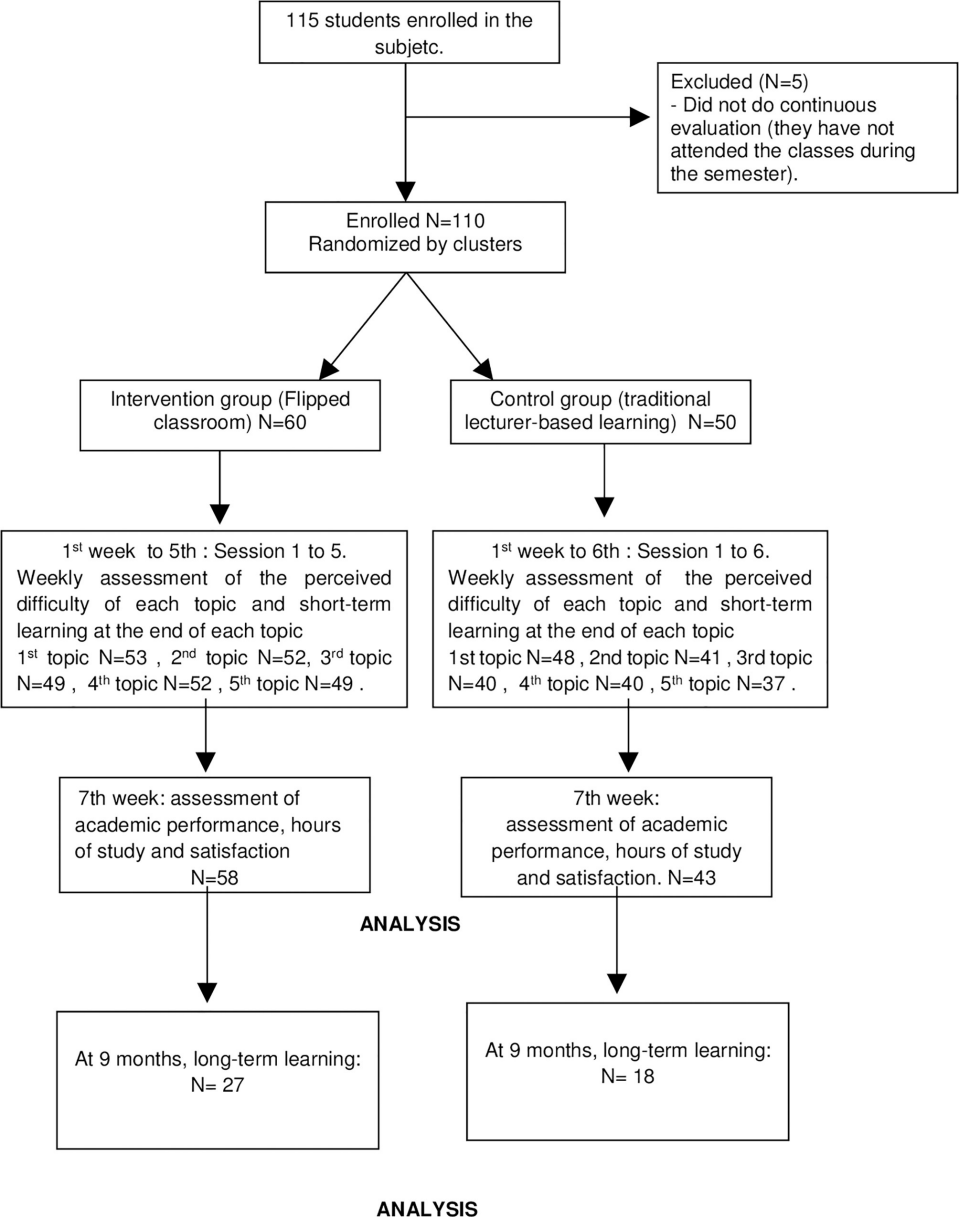
Flowchart.

The main variable and the satisfaction and the hours of study dedicated to preparation for the exam were collected after the seven weeks of teaching. The short-term learning of each theoretical topic and the perceived difficulty of each topic were collected at the end of each theoretical topic. The flowchart and the *n* of each variable are shown in Fig 1.

The following variables were also collected: gender, age, university entrance mark, number of ECTS credits registered throughout the degree and number of credits passed. These variables were collected in order to analyze if the groups of students were equivalent at the beginning of the study.

An analysis of the distribution of the variables was carried out in order to establish the type of statistics to be used; all quantitative variables had a non-normal distribution except for the marks.

It was decided to use non-parametric statistics in the analysis of all quantitative variables with the exception of the variable quantitative grading, which used parametric statistics. Subsequently, a comparison analysis of the groups was performed for the variables gender, age, university entrance mark, number of ECTS credits registered throughout the degree and number of credits passed, to determine if the groups were initially comparable in the study.

Chi-square and Mann-Whitney U statistics were used, depending on whether the variable was qualitative or quantitative. To analyze the effectiveness of FC versus traditional LB learning, both groups were compared with respect to the main variable and secondary variables using the Mann-Whitney U statistic and Student's T based on their distribution.

Statistical analyses were performed with *SPSS 19*.*0* statistical software package, with p-values below 0.05 considered to be significant.

### Ethical aspects

This research project was evaluated and financed by the University of Zaragoza (call for teaching innovation PIIDUZ_1_137). The study has received the approval of the ethical committee of the vice-rector for academic policy of the University of Zaragoza. Approval number–PIIDUZ 17 137. However, the University of Zaragoza has not intervened in the analysis and dissemination of the results. At the beginning of the study, the teaching methodology that was to be developed in each group of students was explained, and all the students gave their verbal informed consent and agreed to participate.

## Results

Initially, a description was made of the variables of gender, age, university admittance mark and credits passed up to the time of the global sample and by groups (to ensure comparability by analyzing the p-value). As can be seen in Table 1, the total sample consists mainly of women (90.1%), with an average age of 20.5 years (SD: 3.27), who entered university to study the degree with an average score of 8.11 (SD: 1.35) and have been approved so far for an average of 57.90 ECTS credits. Table 2 shows that there are no significant differences between the two groups concerning the variables collected. In both tables the values of the mean and standard deviation, median, Q25 and Q27 are shown.

**Table 1:** Description of the variables of gender, age, university entry mark and credits passed up to the time of the global sample.

VARIABLES	TOTAL SAMPLE N = 110			
	Mean (DT)	Median	Q25	Q75
Age	20.5 (3.27)	20	19	21
University entrance mark	8.11 (1.35)	7.90	7.30	9.19
Enrolment credits	61.42 (6.16)	60	60	66
Credits passed	57.90 (6.90)	60	54	60
Gender (%women)	**90.9%**			

**Table 2:** Baseline comparison of the groups using variables of gender, age, university entry mark and credits passed so far.

VARIABLES	FC GROUP N = 60				CONTROL GROUP N = 50				p-value
	Mean (DT)	Median	Q25	Q75	Mean (DT)	Median	Q25	Q75	
Age (mean)	20.75 (4.16)	20	19	21	20.20 (1.69)	20	19	21	**0.729**
University entrance mark	8.34 (1.44)	7.85	7.33	9.30	7.89 (1.25)	8.0	7.0	8.6	**0.423**
Enrolment credits	61.30 (7.28)	60	60	66	61.56 (4.66)	60	60	66	**0.899**
Credits passed	57.71 (4.55)	60	54	60	58.18 (8.87)	60	54	60	**0.968**
Gender (%women)	**91.7%**				**90%**				**0.762**

Mann-Whitney U statistic used in the comparison between groups when showing a non-normal distribution, except in the gender variable, for which the Chi-square statistic was used.

Regarding the comparison of the groups in terms of the academic performance result variable, this was collected by the quantitative grading obtained in the exam. As shown in Table 3, there was a significant difference in both qualitative and quantitative exam scores, with the group that had developed the FC methodology having a higher mean in the exam grade, as well as a higher percentage of students who passed, or received merit and outstanding grades. In addition, in the FC group, exam attendance was greater, since two people failed to take the exam, while in the control group, seven people did not attend.

**Table 3:** Comparison between the FC group and the control group for the outcomes of academic performance (quantitative and qualitative), satisfaction with teaching, study hours for the exam, short-term learning and perceived difficulty variables.

VARIABLES	FC GROUP				LB GROUP				p-value
	Mean (DT)	Median	Q25	Q75	Mean (DT)	Median	Q25	Q75	
Quantitative scores	6.56 (1.58)	6.76	5.42	7.73	5.42 (1.97)	5.24	3.71	6.76	0.002
Satisfaction (quantitative)									
It has promoted new knowledge.	3.05 (0.51)	3.0	3.0	3.0	2.93 (0.60)	3.0	3.0	3.0	0.224
It has favored a deep learning.	2.90 (0.68)	3.0	3.0	3.0	2.95 (0.49)	3.0	3.0	3.0	0.912
It helps critical thinking	2.59 (0.67)	3.0	2.0	3.0	2.56 (0.74)	3.0	2.0	3.0	0.852
It helps applying the theory to practice.	3.13 (0.61)	3.0	3.0	4.0	3.02 (0.82)	3.0	2.0	4.0	0.647
It helps applying theory to the evaluation.	3.10 (0.64)	3.0	3.0	4.0	3.00 (0.74)	3.0	2.0	4.0	0.526
It helps to understand the concepts better.	3.26 (0.54)	3.0	3.0	4.0	3.07 (0.68)	3.0	3.0	3.5	0.252
I think it's a good teaching methodology	3.15 (0.48)	3.0	3.0	3.0	3.10 (0.62)	3.0	3.0	3.0	0.813
Hours of study	14.87 (10.26)	12.0	8.0	20	15.11 (11.56)	10	5.0	25	0.625
Short-term learning									
Topic 1	8.07 (1.41)	8.0	8.0	10	8.5 (1.39)	8.0	8.0	10	0.129
Topic 2	5.49 (1.82)	5.0	3.75	6.25	5.76 (1.93)	6.25	4.43	7.5	0.447
Topic 3	5.50 (1.62)	5.0	4.0	7.0	7.5 (1.66)	8.0	6.0	9.0	0.000
Topic 4	7.14 (1.86)	7.0	6.0	9.0	8.25(1.77)	9.0	8.0	9.0	0.002
Topic 5	7.11 (1.27)	7.4	6.0	8.0	6.92(0.03)	6.6	6.6	7.4	0.175
Long-term learning (eight months)	6.41 (1.57)	7.0	5.0	7.0	6.78 (1.70)	7.0	6.0	8.0	0.220
Perceived difficulty									
Topic 1	5.37 (1.92)	6.0	4.0	7.0	2.26 (2.01)	2.0	1.0	3.0	<0.001
Topic 2	5.29 (2.13)	6.0	4.0	7.0	5.90 (1.62)	6.0	5.0	7.0	0.342
Topic 3	5.96 (1.52)	6.0	5.0	7.0	6.61 (1.65)	7.0	5.0	8.0	0.057
Topic 4	6.27 (1.40)	6.0	5.0	7.0	6.59 (1.67)	7.0	6.0	8.0	0.269
Topic 5	5.37 (1.92)	6.0	4.0	7.0	6.68 (1.77)	7.0	6.0	8.0	0.002
Qualitative scores									
Outstanding	6.9%				2.3%				
Merit	34.5%				20.9%				0.025
Pass	39.7%				30.2%				
Fail	19.0%				46.5%				

Statistics used: Student’s T to analyze the quantitative marks variable, Chi-squared to analyze the qualitative marks variable, and the Mann-Whitney U statistic for the rest of the variables.

Regarding the comparison of the groups in the "satisfaction" variable, the results obtained show that there were no significant differences in satisfaction between both groups of students, obtaining in general a good result in both groups. Regarding the hours dedicated to the study of the subject for the exam, there was no significant difference between the two groups. There were significant differences in the perceived difficulty and short-term learning of some topics, with the FC group finding them less difficult with the exception of the first topic, but there was also worse short-term learning in some subjects in the FC group.

Regarding the qualitative assessment of student satisfaction, both groups considered that the theory of the subject *Social Work with Groups* is interesting, and it was imparted in a dynamic way, but in the group of students who had followed the FC methodology, they added a greater percentage of comments on the effectiveness of the teaching methodology that, according to their criteria, helped them to understand, further their knowledge and update themselves on the subject.

## Discussion

In relation to the hypotheses proposed at the beginning of the study, it has been verified that the students who developed an active methodology of FC-based learning achieved a higher academic performance (quantitative and qualitative), and a lower perception of difficulty of the content with respect to the students who developed their learning through a traditional methodology. But it was not shown that the students who developed an active methodology of FC-based learning took greater satisfaction from the subject and the methodology used, achieved better long-term learning or that they spent less time in preparation for the exam.

Regarding the academic performance variable, the results obtained are in line with the existing bibliography. A meta-analysis that analyzed 41 studies comparing the effectiveness in the examination score of FC versus LB, found no statistically significant differences in five studies (with values of standardized mean difference between -0.66 and -0.02) but in the rest of the 36 studies, there were statistically significant differences (with values of standardized mean difference between 0.09 and 2.49).

The total value of the standardized mean difference obtained was 0.47 (CI: 0.31, 0.63). It must be taken into account, however, that of the 41 studies reporting examination scores, seven were before-after studies and provided pre-post score changes. In Chen's study [10], all examination score outcomes were evaluated according to Kirkpatrick's level 2 measurement (learning) [34], but in this study, level of change was additionally evaluated. Studies in social work [30–31] did not assess academic performance specifically.

The Holmes study [31] was conducted with master’s students, and they used Google for teaching resources, while the study by Sage & Sele [30] evaluated student preparation and engagement through a survey.

With regards to the study time used for preparation for the exam, no statistically significant differences were found, since the students invested an average of 14.87 (SD: 10.26) and 15.11 (SD: 11.56) hours in the FC and LB groups respectively. However, taking into account the results obtained in the grading, it could be said that with the same study time, the students who followed an FC methodology achieved a better grade, results that are in line with the Baepler et al. study [24]. On the other hand, it could also be considered that social desirability has influenced student responses.

The satisfaction and positive feelings towards this teaching methodology and against LB have been demonstrated in several research studies [19, 35–37], but there are also studies in which student satisfaction does not differ between both teaching strategies [38–39].

Among the reasons found in the literature, higher workload stands out [38], although in this study, to minimize the bias of the extra workload, students who followed the FC methodology had two hours less class. The explanation in our study can be linked to the fact that in both groups a high satisfaction level was obtained (scores of around 3 points out of 4 in all items), since, according to students' feedback generally and over a number of years, they consider the subject of *Social Work with Groups* to be interesting and show high motivation towards it, besides considering it useful for their professional future. With regard to short-term learning, significant differences were obtained in two of the five subjects studied, obtaining a worse score in the FC group, with results contrary to those that might have been initially predicted. This may be because topics 3 and 4, the subjects in which a significant difference was obtained, are those that may be considered more difficult or with a larger content, since they are related to the theory of the group structure that covers the concept of structure, status, roles, norms, and group culture, and which contain theories of leadership. It may be that when the content is complicated, more time needs to be invested in class to correctly assimilate the contents, or a mixed methodology of FC and LB needs to be developed. Regarding long-term learning, this study found that both groups obtained results without significant differences.

A study by van Vliet et al. [40] evaluated the effect of the FC teaching methodology after five months with respect to the variables of learning and motivation strategies using the Motivated Strategies for Learning Questionnaire, and compared to the traditional methodology, obtaining results that the values of critical-thinking, task value, and peer instruction had decreased to similar levels of those in the traditional learning group.

Results in this study coincide with those of the study carried out by van Vliet et al. [40]. Learning was assessed through a test-type questionnaire having three response options but different from those used to evaluate academic performance, and it was found that learning was maintained equally in both groups. However, these results should be viewed with caution since the number of students taking the test at eight months was small in both groups. Furthermore, having viewed the results, it is possible that the test did not sufficiently distinguish between the level of in-depth learning, since ten questions were used that included both basic and fundamental theoretical content aspects. Perhaps a more complex test or more complex questions would have allowed for the discerning of more in-depth learning.

The first topic, which addresses the definition of groups and types of groups, could be considered to not involve too much difficulty, but the FC group perceived it to be significantly more difficult. This may be due to the fact that the learners were initially being exposed to the concept of flipped classrooms and as the onus on learning was shifted towards them, they perceived the workload to be greater, or they were anxious about potential classroom disruption [28, 33, 41]. Once the FC dynamic was established and they adapted, in the rest of the topics, the perceived difficulty was always greater in the LB group, becoming significant in the last topic.

This study presents both strengths and limitations. Among the strengths can be highlighted the contribution of this study to evidence concerning this active teaching methodology in the social sciences, since studies are scarce in these disciplines, as well as the methodological rigor, since a randomized methodology was developed using clusters with two groups in parallel. This methodology provides great scientific evidence if all biases have been controlled [42].

In addition, to ensure the aforementioned methodological and intervention quality, the recommendations of Rotellar & Cain [33] were followed. And finally, secondary variables were collected, both from study hours and from the effect of short-term and long-term learning that may shed light on the effectiveness of this teaching methodology compared to the traditional LB methodology.

On the other hand, this study also has certain limitations. The main limitation lies in the fact that the study was carried out in one university, with only two class groups, which were randomized. Although these groups are comparable in sex, age, credits registered for and received and degree entrance score, there may be other confounding factors that were not considered, such as income and work history. At the University of Zaragoza (Spain) in the 2017–2018 course year, three student groups took the Social Work with Groups course, distributed over two morning groups and one afternoon group. It was decided that the afternoon group would not be included in the randomization, mainly because this course was taught by a faculty member that was different from the one teaching the morning groups and also because the student profile for this group is different, having a higher percentage of older students and individuals that work during the morning hours. Therefore, it was believed that significant biases could possibly arise if this third group was included in the study.

Another limitation of this study lies in the assessment of the long term learning (at 8 months), since the sample size of the participants in this assessment was small and a large percentage participated anonymously. Therefore the data has been analyzed as a group (mean, median and quartiles). Another limitation is the sample size. The sample consists of 110 subjects, but in order to establish greater scientific evidence, it would be necessary to increase the sample size. Another potential limitation is that the evaluator who assessed the study variables was not blind to the assignment, although he/she could not interfere in the results, since they were collected from a test or using a numerical scale ranging from 1 to 4 or 0 to 10. And finally, the social desirability bias may have influenced the measurement of the hours of study preparing for the exam variable.

## Conclusions

The FC teaching methodology in comparison with the LB methodology has shown itself to be a more effective tool regarding academic performance evaluated in a quantitative and qualitative way with regards to Social Work education at university level. It has also been evaluated more positively in terms of the perception of difficulty of the content. However, no significant differences have been found regarding satisfaction with the subject and the methodology used, long-term learning and the time spent preparing for the exam.
